# Molecular Codes in Biological and Chemical Reaction Networks

**DOI:** 10.1371/journal.pone.0054694

**Published:** 2013-01-23

**Authors:** Dennis Görlich, Peter Dittrich

**Affiliations:** 1 Bio Systems Analysis Group, Institute of Computer Science, Jena Centre for Bioinformatics and Friedrich Schiller University Jena, Jena, Germany; 2 Institute of Biostatistics and Clinical Research, University of Muenster, Muenster, Germany; Michigan State University, United States of America

## Abstract

Shannon’s theory of communication has been very successfully applied for the analysis of biological information. However, the theory neglects semantic and pragmatic aspects and thus cannot directly be applied to distinguish between (bio-) chemical systems able to process “meaningful” information from those that do not. Here, we present a formal method to assess a system’s semantic capacity by analyzing a reaction network’s capability to implement molecular codes. We analyzed models of chemical systems (martian atmosphere chemistry and various combustion chemistries), biochemical systems (gene expression, gene translation, and phosphorylation signaling cascades), an artificial chemistry, and random reaction networks. Our study suggests that different chemical systems posses different semantic capacities. No semantic capacity was found in the model of the martian atmosphere chemistry, the studied combustion chemistries, and highly connected random networks, i.e. with these chemistries molecular codes cannot be implemented. High semantic capacity was found in the studied biochemical systems and in random reaction networks where the number of second order reactions is twice the number of species. We conclude that our approach can be applied to evaluate the information processing capabilities of a chemical system and may thus be a useful tool to understand the origin and evolution of meaningful information, e.g. in the context of the origin of life.

## Introduction

In recent years great advances have been made in understanding the biochemical basis of biological information processing. For theoretical analysis of biological information Shannon’s theory of communication [1] has been applied very successfully in various domains, like gene regulatory networks [2], bacterial quorum sensing [3], or signaling in molecular systems [4], [5]. The mathematical theory of communication focusses on uncertainty of events and intentionally neglects semantic aspects of information, because “*they are irrelevant for the engineering problem*” (Shannon [1], p. 1). However, in order to obtain a full understanding of biological information, studying also semantic as well as pragmatic aspects would be important, if not necessary [6], [7]. Although syntax, semantics, and pragmatics are interdependent [8], we focus here only on the semantic aspects of molecular networks in order to keep our formalism and analysis clear and concise.

In general, semantics refers to the relation between a sign and its meaning. This relation can be characterized by a code, which is a mapping from the signs to their meanings [9]. For example, the genetic code is a mapping between codons and amino acids [10], which is realized in cells by a complex translation machinery. An important property of a code is its contingency. This means that the relation between signs and meanings could be different, thus the relation is not determined by the signs and meanings alone [6], [9]. In particular, this implies that natural laws allow to derive the relation only by knowing the context under which the signs are interpreted.

Furthermore, it implies the existence of another context under which the signs are interpreted differently. This is why we say that the relation between signs and meanings, i.e. the code, cannot be explained by physical laws [11], like the natural laws do not help in understanding the written law or the grammar of a language. However, this notion of independence from natural laws sometimes causes confusion [11].

In order to properly use semiotic concepts in biology we should provide a link to the realm of physics by (1) selecting an experimentally grounded and reliable formal description of the targeted biological system, by (2) providing precise, not necessarily formal, definitions of the semiotic concepts that shall be applied to the system, and by (3) interpreting these definitions by linking them to the formal description of the biological system. (1) We use reaction networks as a formal description, (2) link it to the notion of organic codes as reviewed by Barbieri [9] and (3) develop a formal definition of a molecular code with respect to reaction networks [12].

With this approach, the semiotic concept of code gets – at least partially – operationalized by means of physical experiments. In particular, it allows to incorporate contingency in a formal model of molecular codes.

**Figure 1:**
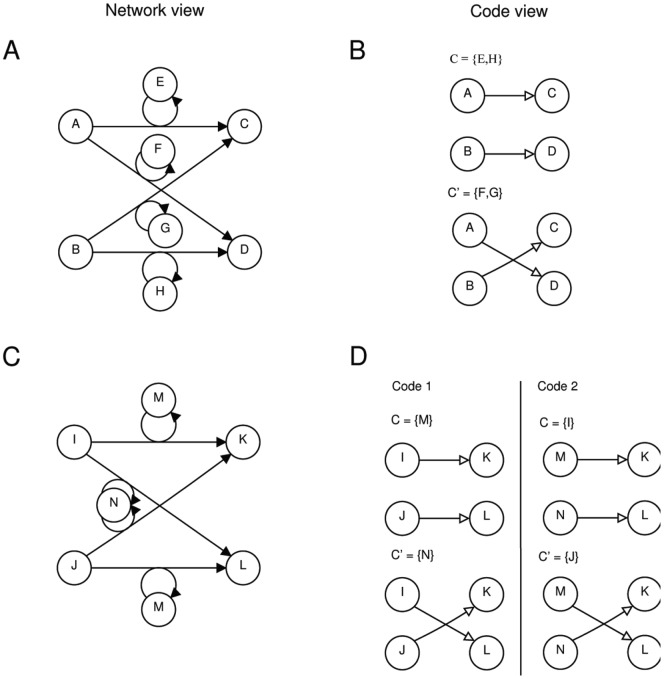
Two exemplary reaction networks containing molecular codes. Panel A: Chemical reaction network 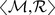 with species 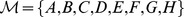 and reaction rules 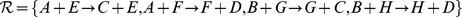; panel B: Code pair that can be realized by the network in panel A. The binary molecular codes are characterized by 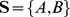, 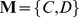, and the two molecular contexts 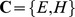, and 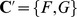; panel C: Chemical reaction network with species 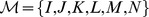 and the reactions 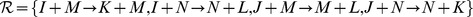; panel D: Two molecular code pairs can be realized by the network in panel C. Note that our code analysis does not depend on catalysis. Replacing a reaction like 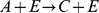 by 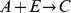 would lead to the same molecular codes.

To illustrate the basic idea we will briefly discuss an example reaction network that contains a contingency. Fig. 1A shows a reaction network containing eight molecular species and four reactions. We assume that the network contains all possible reactions that can appear when mixing these molecules. The network then is assumed to be a complete model of the world, i.e. no species and reactions are missing that are physically possible. A reaction network can implement a *mapping* among molecular species. Here, for example, 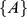 can be mapped to 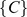 by reaction 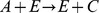. 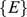 is necessary for the reaction to happen and thus we call it a *molecular context*. The network can implement a *molecular code*, if there exists a set of molecular species that can be mapped on a second set of molecular species in at least two different ways. In this example network the sets 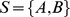 and 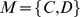 fulfill this property. **S** (*domain*) maps to **M** (*codomain*) by applying the context 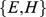. No two elements of the domain **S** map to the same element in the codomain **M**. There exist an alternative molecular context 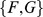, which realizes a different mapping between domain and codomain, so the mappings qualify as molecular codes.

## Methods

In this section we provide a formal definition of a molecular code as a contingent mapping with respect to a reaction network. Then we formally define a reaction network’s semantic capacity based on the number of molecular codes it can realize, and finally describe two algorithms for identifying molecular codes in a reaction network.

### Molecular Codes are Contingent Molecular Mappings

A *reaction network* 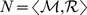 is defined by a set of molecular species 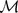 and a set of reactions 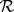 occurring among the molecular species 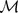. See Fig. 1A for an example. For each reaction 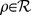, let 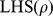 and 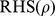 denote the set of reacting and produced species of reaction 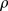, respectively.

A subset of molecular species 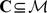 is *closed*, iff the application of all possible reactions from 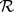 on **C** does only produce species from **C**, i.e. for all 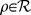 with 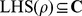: 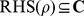 [13]. For every set of species 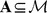 there exists a smallest closed set 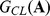 containing **A** [14]. We say that 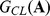 is the *closure* of **A**. Intuitively, the closure of a set of species contains all those species that can be reached by an arbitrary long reaction path among the species of **A**.

Given a reaction network 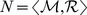 and two sets of molecular species 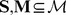, we say that 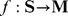 is a *molecular mapping* with respect to *N*, iff there exist a set of species 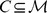 (called context), such that for each pair 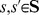 with 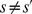: 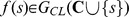 and 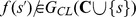. If there exists a molecular mapping *f* with respect to *N*, we also say that *N* can *realize* the molecular mapping *f*.

Note that in a reaction network there is usually more than one molecular context **C** that realizes a particular molecular mapping *f*. Intuitively, in order to “compute” 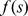 with the reaction network *N*, we put all molecules from the context **C** together with *s* in a reaction vessel. Then we repeatedly apply all applicable reaction rules and add the products to the reaction vessel until no novel molecular species can be added anymore. Then we check which molecular species from **M** is present, which must be – according to our definition – only one species and the result of 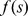.

Given a reaction network 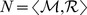 and a non-constant (A mapping 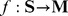 is called non-constant, iff there exists 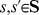 such that 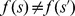) molecular mapping 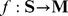, with 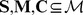 we call the mapping 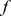 a *molecular code* with respect to 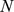, if all other mappings 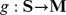 with the same domain **S** and codomain **M** can also be realized by the reaction network 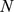, i.e. there exist alternative molecular contexts to map **S** to **M**.

The definition catches the notion of contingency as described above, i.e. the elements of the domain can be mapped to the elements of the codomain in a contingent way by changing the molecular context. In a semiotic interpretation we can also say domain and codomain contain the signs and meanings, respectively. The molecular context thus becomes the “codemaker”, i.e. it is necessary to realize the code. In general, the definition given above allows for codes of arbitrary size. In order to keep our study tractable, we will focus on molecular codes that are binary, i.e. where **S** as well as **M** contain exactly two molecular species [12]. We will also not study molecular mappings that are only partially contingent. For binary molecular codes our definition can be reformulated as follows:

Given a reaction network 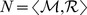 and two binary sets of molecular species 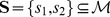 and 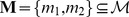. The mapping 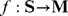 is called *binary molecular code* (BMC), iff there exist two sets 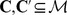, such that the following conditions hold:
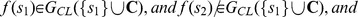


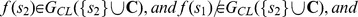


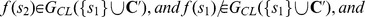


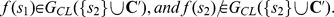



Each binary molecular code comes with a second code implementing a different mapping. The alternative code 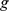 is determined by 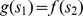 and 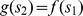. 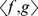 is called *code pair*. Two simple example networks are shown in Fig. 1A and 1C (cf. Dataset S1 and Dataset S2 for the network description). Both networks appear to be very similar in their structure, but contain different numbers of code pairs. While the former network is capable to realize one code pair, the latter network – though being smaller – can realize two code pairs.

### A Network’s Semantic Capacity can be Measured by Molecular Codes

A system’s *semantic capacity* 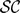 is its ability to realize contingent molecular mappings, i.e. the number of code pairs 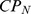 that can be identified,
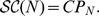



To compare different semantic capacities we can also use the *logarithmic semantic capacity*

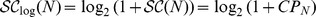
especially with very high values of 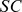. We apply the transformation 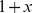 to guarantee that 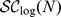 is well defined and its smallest value is zero, in case the network cannot realize any molecular code.

In future studies, the semantic capacity can be integrated with measures of the code’s quality, fitness, or cost [15], [16]. e.g. two networks with the same number of code pairs could be differentiated with respect to the costs to implement those codes.

### Molecular Codes can be Identified Algorithmically

The formal definition of binary molecular codes allows to develop code-identifying algorithms. In general, the algorithms search for a combination of molecular species and reactions fulfilling the BMC conditions. Different approaches can be used to implement the BMC conditions, i.e. via closed sets, or via paths.

The closure-based algorithm calculates all closed sets and checks combinations of six closed sets for the BMC conditions. In particular, for the two elements of the domain, and the two elements of the codomain the single molecular closed sets, i.e. the closed sets that are generated by a single molecular species alone (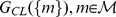), are used. There exist at most 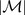 single molecular closed sets. The closure-based algorithm has a worst-case running time complexity of 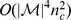 with 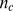 as number of all closed sets contained in the system.

Domain and codomain are connected by reactions such that an alternative algorithm can be formulated using the network’s paths. For the identification of BMCs the paths for all pairs of species are identified. Every combination of four paths is checked for the BMC condition. The running time complexity of this *path-based* algorithm depends on the number of paths the network contains, which can grow enormously with the network’s density. Therefore, we apply a parameterized algorithm that uses only the 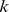-shortest paths [17] between every pair of species. The worst case running time of the parameterized algorithm is bounded by 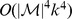. If 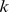 is chosen too small the algorithm is not able to find all codes in the system, but gives an approximate measure. Large values of 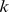 resemble the non-parameterized path algorithm, since all paths are considered for the analysis. Pseudocode for the parameterized path algorithm, the closure-based algorithm and subroutines is given in Text S1. The different running time complexities suggests a conditional application of the algorithms. The path-based algorithm can be efficiently applied on networks that have a high number of closed sets and a low number of paths, while the closure-based algorithm can be applied in the other case, where the number of paths is high and the number of closed sets in the network is low. Interestingly, systems with high semantic capacity tend to have both, high number of closed sets and many paths, such that an algorithmic challenge remains for analyzing such systems.

## Results

We survey different kinds of systems for their semantic capacity by the application of the algorithms described above. In particular we analyze the gene translation chemistry, gene regulatory networks, phosphorylation cascades, combustion chemistries, the martian atmosphere photochemistry, and random reaction networks. As a result of the analysis we can assign semiotic roles to the molecular species. Table 1 summarizes the semiotic structure of the analyzed biological systems. For details on all analyzed networks see Table S1.

**Table 1:** Overview of semiotic interpretation of the biological systems surveyed.

Role	Gene regulatory codes	Genetic codes	Phosphorylation cascade codes
Signs	transcription factor s	DNA codons and/or unloaded tRNAs	high concentration of kinases and/or phosphatases
Meanings	gene product s	amino acid s	high/low concentration of target molecules
Molecular contexts	DNA with promoterand coding region	loaded tRNAs or a combination of loaded tRNAs, aaRSs, and codons	kinases and/or phosphatases

### The Genetic Code is a Molecular Code

The genetic code, i.e. the mapping describing the translation from nucleotide triplets to amino acids, was the first biological code described as such [18] and is often used as initial example for molecular codes [9], [15], [19].

To check whether the genetic code is a molecular code as defined in this paper we need to identify contingent molecular mappings in the reaction network describing the translation from codons to amino acids. In recent species only one code is realized, thus the reaction network taken from a certain species will not contain any molecular codes. A reasonable approach to overcome this effect is to merge the known genetic codes in one reaction network, such that the merged network contains all (known) alternatives. Note that merging two chemical networks has to be done carefully to avoid unwanted inconsistencies. In particular, the networks to be merged needs to be from the same physicochemical context, which determines the reactions of the network model. This guarantees that no “artificial” contingencies are introduced. The gene translation chemistries studied here can be merged, because they take place in the same environment.

The fact that there exist more than one genetic code is known for a long time [20], [21]. The 17 known genetic codes, as listed at NCBI [22], cover nuclear and non-nuclear codes of different genera, e.g. bacterial, archaeal, and plant plastid codes, the vertebrate, invertebrate and yeast mitochondrial codes, and the alternative yeast nuclear code. The flexibility of the genetic system is also underlined by the possibility to introduce even unnatural amino acids to the genetic codes of various organisms [23]. For our analysis, we merge the 17 codes listed at NCBI by constructing a reaction network containing the 64 codons, 20 amino acids, and the specific tRNAs, which are necessary for the translation. For all mappings between DNA triplets and amino acids occurring in the 17 codes we add a reaction in the network of the form 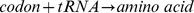 (see Dataset S3).

The algorithmic analysis of this network identified 16 binary molecular codes (see Text S2 for a complete list), i.e. a logarithmic semantic capacity of 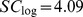. The binary codes can partly be assigned to larger molecular codes. For instance, the codons CTT,CTG,CTA, and CTC can be mapped on leucin (L) and threonin (T) and give rise to six of the found BMCs. A second group involves the mapping between AGG,AGA and glycin (G), serine (S), arginine (R) and the translation stop. This code can also be decomposed into six BMCs. There does exist four more BMCs that involve the codons TCA, TTA, TAG and TAA and the amino acids leucine (L), glutamine (Q) and the stop signal. The data suggests that it is easier for the cell to change the mapping for the stop signal, than for an amino acid. Table 2 summarizes the identified BMCs. The general existence of alternative mappings in the genetic translation system suggests that the genetic code qualifies as a molecular code. The relatively small semantic capacity of the merge network demonstrates that the genetic code, thus a principally contingent system, is under strong constraints, regarding the assignment between codons and amino acids.

**Table 2:** Molecular codes in the reaction network model of the 17 known genetic codes.

Signs (codons)	Meanings (amino acids)	#BMC	References
CTT, CTG, CTA, CTC	L, T	6	[20], [24]
AGG, AGA	G,S,R, Stop	6	[20], [25]–[36]
AGG, TCA	S, Stop	1	[20], [27], [28], [31], [33], [37]
AGA, TCA	S, Stop	1	[20], [27], [28], [31], [33], [37]
TTA, TAG	L, Stop	1	[20], [22], [37]–[39]
TAA, TAG	Q, Stop	1	[20], [40]–[43]

Here the 16 BMCs found in the merge of the 17 known genetic codes are summarized. If applicable BMCs are grouped. References: Articles reporting the respective alternatives in the genetic code that are part of a BMC in this analysis.

To calculate the system’s potential maximum semantic capacity we extend the reaction network model by including all potential mappings between codons and amino acids even if they have not been observed so far. The model includes all possible tRNA molecules, such that each codon could be read for each amino acid. In such a system the number of binary molecular codes can easily be calculated. Each pair of codons forms a code pair with each pair of amino acids. Since there exist 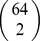 pairs of triplets and 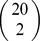 pairs of amino acids the number of BMCs is
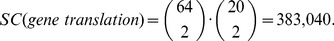
(1)


The logarithmic semantic capacity is approximately 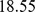. The difference to the merge network (which relies completely on observed variation in the code) suggests that cells use only a small fraction of their semantic capacity.

**Figure 2:**
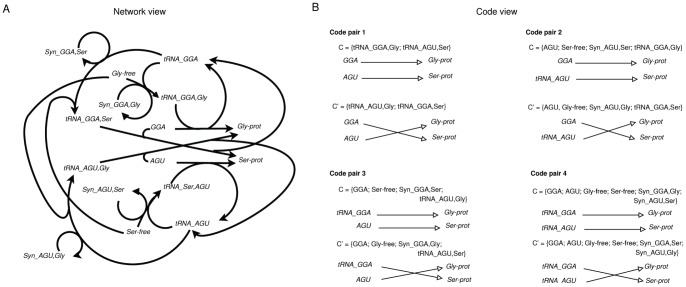
Subnetwork of the full gene translation network model with synthetases (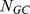) and the realized molecular codes. The network (panel A) shows a subnetwork of the gene translation network model containing the translation, and loading reactions for two selected codons (GGA, AGU) and amino acids (Gly, Ser). The semantic analysis shows that four code pairs can be implemented by this network (panel B).

The analysis of molecular codes relies on the identification of the adapters [9]. In the two models above the tRNAs are the adapters and carry the combinatorial complexity of the system. In the following we analyze a more realistic model of the gene translation machinery by including the loading step of the tRNA. The refined network model 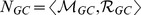 contains all possible mappings between the 64 codons and 20 amino acids as described above. Additionally, we model the loading step of the tRNAs by inserting the respective amino acyl tRNA synthetases (aaRS) (cf. Fig. 2). The reaction network 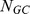 describes the core molecular mechanism realizing the standard genetic code and all alternative codes. The set of molecular species 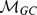 contains all DNA strings of length three (Table S2, Eq. 2), representing the codons, the twenty proteinogenic amino acids in their free form (Table S2, Eq. 3), the twenty amino acids bound in a protein (Table S2, Eq. 4), all possible tRNAs in their unloaded (Table S2, Eq. 5) and loaded form (Table S2, Eq. 6) and all possible aaRS (Table S2, Eq. 7), such that the system is able to load all amino acids to all tRNAs.

The set 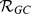 contains all reactions loading the amino acids onto the tRNAs (Table S2, Eq. 8) and all reactions inserting an amino acid in the peptide sequence (Table S2, Eq. 9). Fig. 2A displays a subnetwork (Dataset S4) with two codons (GGA, AGU), two amino acids (Gly, Ser) and the respective other elements of the network (tRNA and synthetases).

Analyzing the subnetwork (Fig. 2, Dataset S4 ) allows to assess the whole network’s semantic capacity. Table 3 shows the four contained molecular code pairs, the respective molecular contexts are listed in Table 4. The identified code pairs (Table 3) show that not only codons can be signs, but also the unloaded tRNAs can function as signs. These additional signs increase the number of code pairs in a combinatoric manner. The “new” codes differ structurally in their molecular context. While, classically, the codons are mapped to the set of amino acids using the loaded tRNAs as context, the new signs, i.e. unloaded tRNAs, are mapped to the set of amino acids by using a molecular context that consists of the free amino acid loaded to the free tRNA, the synthetase performing the loading step, and the codon that needs to be recognized by the tRNA. The number of code pairs in this system can be calculated by
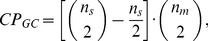
(2)with 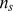 as number of signs and 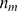 as number of meanings (amino acids). For the full gene translation system the number of signs is 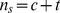, with 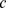 as number of codons and 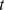 as number of unloaded tRNAs. Since there is always one pair of one tRNA and codon belonging together, which therefore can not be combined in an BMC, we have to subtract the number of such pairs 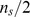 from the amount of all combinations.

**Table 3:** Code pairs in the gene translation model.

Code pair	Signs	Meanings
1	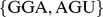	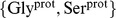
2	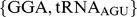	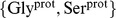
3	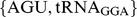	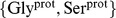
4	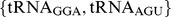	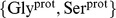

Code pairs realized by the subsystem of the gene translation network with synthetases shown in Fig. 2.

**Table 4:** Molecular contexts of the codes in the gene translation model.

Code pair	Molecular context	alternative molecular context
1	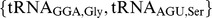	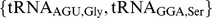
2	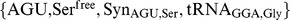	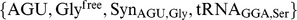
3	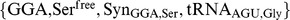	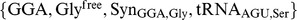
4	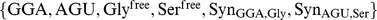	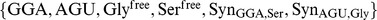

Molecular contexts of the code pairs shown in Table 3.

Using Eq. (2) t he analysis of the whole network (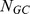), describing all potential genetic codes with 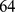 codons and 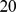 amino acids, results in 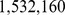 binary code pairs, i.e. 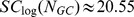. This is a different result than for the less detailed model, as calculated by Eq. (1). The extension of the model by aaRS, unloaded tRNAs, and unloaded amino acids increases the semantic capacity. This increase is not only an artifact from increasing the network size, but results from qualitative new code pairs.

The question to what extend a tRNA based code could be employed by the cell is open, but the potential existence of such a code is nevertheless an interesting result.

### Gene Regulation by Transcription Factors Allow for Molecular Codes

In general, the gene regulatory network (GRN) of a cell constitutes the regulatory relations between genes. A particular regulatory relation is a fairly complex process involving a gene, the promoter and binding region of that gene, the binding of the transcription factor (TF) plus c ofactors, and the production of a product by the recruitment of the gene expression machinery. We will show here that a cell’s GRN is also a highly semantic system.

**Figure 3:**
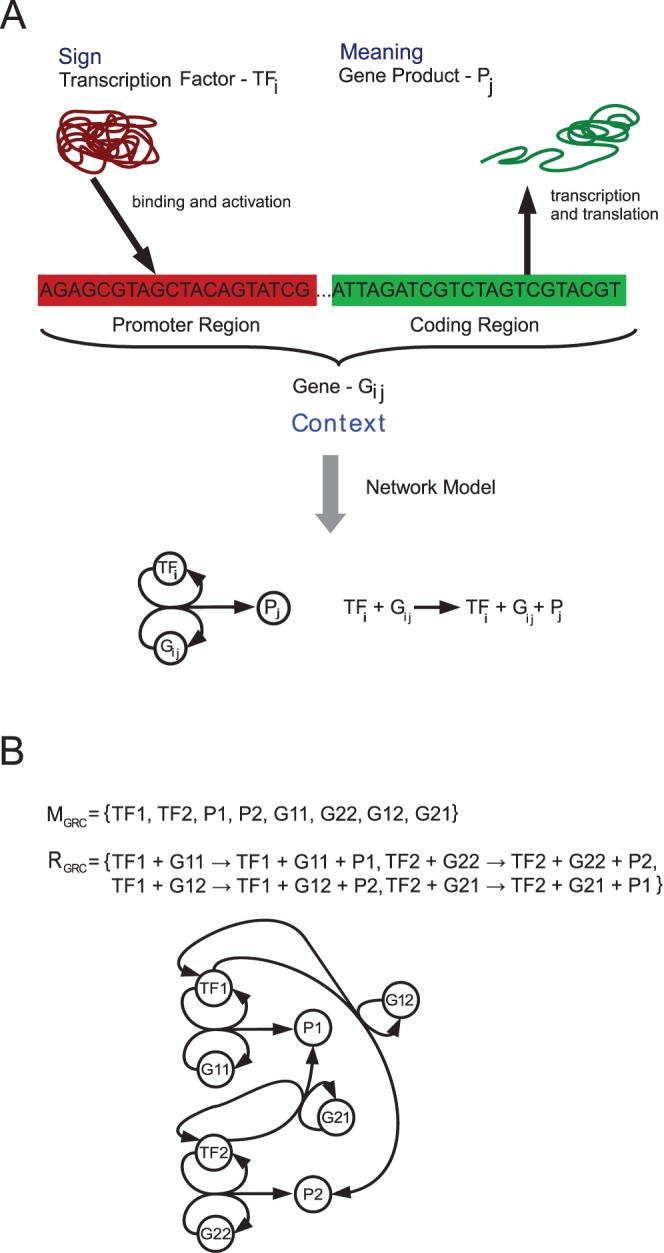
Gene regulatory network model. Panel A: Model of the expression of a gene, and the reaction network formulation of the same process (below). Blue text in panel A indicates the semantic interpretation, i.e. the transcription factors are the signs, the products are the meanings, and the DNA is the molecular context. Panel B: reaction network constructed according to the formalization of gene regulation shown in (A) containing two transcription factors (TF1, TF2), two gene products (P1, P2) and the according genes (G11, G12, G21, G22).

In order to do so, we model a GRN as a reaction network 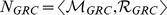 by explicitly inserting the relevant components (Fig. 3). The resulting network is not a generic model to describe all possible gene regulatory networks, but a model that covers the main properties of regulation important for this study. 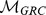 contains 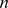 transcription factors 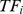, 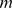 products 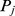, and genes 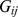. Each gene 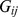 represents a combination of a promoter site 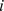 and a coding region 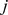, where the promoter site 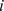 is specific to 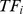 and the coding region 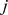 produces 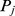. For our model we assume that there exist as many promoter sites and coding regions as transcription factors and products, respectively, such that each promoter-gene combination is possible. In summary
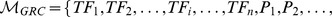


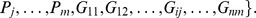



Note that the differences of eukaryotic and prokaryotic gene regulation are abstracted by our model, because only the general mechanism of transcription factor regulated expression that gives rise to a high semantic capacity shall be explored here. Therefore, we consider transcription factors that bind only one promoter and that a promoter is bound by only one transcription factor. Then, the expression of a gene 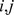 is given by
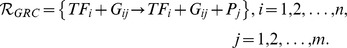



The semantic analysis shows that the reaction network can implement molecular codes, but only in one way, i.e. with the transcription factors as signs and the set of products as meanings. The set of genes, i.e. the combination of promoter and coding region, forms the molecular context. So the mapping between transcription factor and gene product can be altered by the exchange of a promoter region of a gene (or vice versa). Such promoter exchanges are also a common tool in molecular biology to allow for the external control of gene expression [44], e.g. to discover the function of silenced gene clusters [45].

Interestingly, in contrast to the model of the gene translation chemistry described above, the DNA is not the sign, but functions as the molecular context. This “role change” suggests an interdependence between different codes. Here the “gene regulatory code” regulates the execution of the “gene translation code”, as the former one controls the usage of the latter’s signs.

Note that the reaction network model can easily be made more complex by modeling transcription factors as protein complexes and including the respective assembly processes, by modeling different types of transcription factors (activators, repressors, enhancers), or the introduction of several DNA binding sites in the regulatory region to allow a combinatoric regulation by several transcription factors. However, the general conclusion about the semantic capacity of a GRN would not be affected.

### Signaling by Phosphorylation Cascades Allows for Molecular Codes Only in a Dynamic Setting

Cells maintain different systems for signal transmission and integration [46]. The most prominent signaling systems rely on reversible phosphorylation of amino acids side-chains for regulation of signaling protein activity. The direct involvement of such systems in signaling suggest that they may be semantic systems. If so, they should be able to realize molecular codes. We have studied phosphorylation cascades, like the mitogen activated kinase regulatory network, as a typical instance of an intra-cellular signaling system. These systems demonstrate the limitation of our static approach. Here, it is necessary not only to distinguish between molecular species, but also between their concentrations. By assigning concentration levels to each species we allow for the dynamic change of these concentrations by the system’s reactions. Thus, a molecular species’ concentration is decreased if it is used as reactant in a reaction and increased if produced by a reaction. A species can have an effect on another species’ concentration through the reactions in the system.

**Figure 4:**
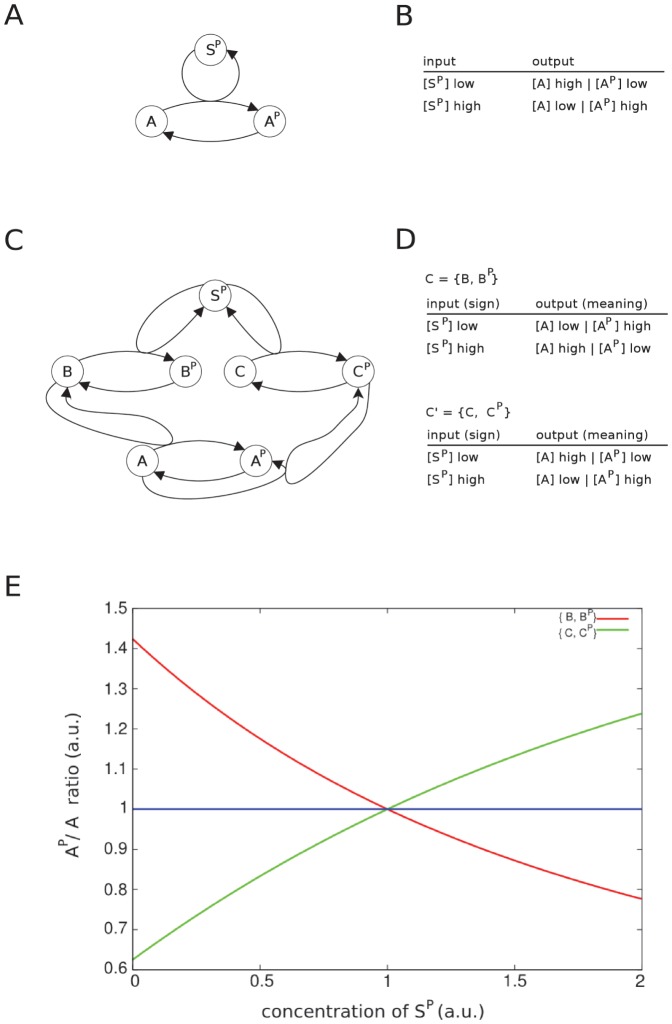
Reaction networks describing phosphorylation motifs. Molecular species in these networks represent kinases that may be activated or inactivated by phosphorylation. Activated and non-activated forms of a kinase are modeled as different species (e.g. species 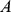 and 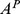). Panel A: Reaction network of a simple phosphorylation motif, which can realize a molecular mapping, but not a molecular code. Panel B: Molecular mappings that can be realized by the reaction network from panel A. These mappings do not constitute a molecular code. Panel C: M ore complex reaction network that can realize molecular codes. Panel D: The two binary molecular codes (i.e., one code pair) are realized by either one of the two molecular contexts 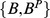 or 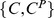. In contrast to the other described molecular codes (e.g. the genetic code), here, the code is not only specified by the species also, but also by the species’ concentrations. Panel E : Simulation of the second network (panel C) showing the 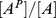 ratio over 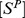 for the two different contexts. The red line shows the system’s behavior for the context 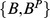, while the green line shows the system’s behavior for the alternative context 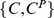 over varying initial concentrations for 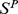. The blue line indicates the (here arbitrary) threshold to separate high and low concentrations.

In general, the activation of a kinase by phosphorylation can generate a molecular mapping between the kinase and its target, but this mapping is not necessarily a molecular code (Fig. 4A). In contrast, a two-step cascade is able to implement a molecular code (Fig. 4C).

The simple one-step phosphorylation model (Fig. 4A) contains two kinases: an initial kinase (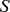) and a target kinase (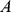) which can be phosphorylated by 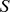 (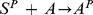). We also model the dephosphorylation (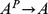). For sake of simplicity we do not model the phosphatases, and the phosphate related molecular species (e.g. ATP, ADP, P) involved in the process, but assume a buffered concentration. In the simple one-step model we can identify a molecular mapping between 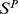 and the two states of kinase 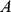 (Fig. 4B). If 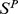 has a low concentration the system is in a state where the unphosphorylated state 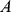 has a high concentration and the phosphorylated state 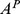 has a low concentration. According to the definition of molecular code given above the system should be able to change the mapping, i.e. be contingent, by the application of a different molecular context to realize a code. Here, no alternative mapping between 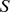 and 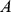 can be realized, such that the system is not able to realize a molecular code.

If we consider a different system where two kinases are between 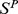 and 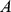, we obtain a two-step phosphorylation cascade (Fig. 4C). 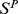 now phosphorylates the inserted species, while these have an effect on 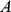. The system has the possibility to “choose” between two alternative systems, i.e. the inserted species may be “active” in the unphosphorylated state (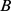), or in the phosphorylated state (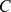). There exist several mappings in such a system, e.g. between 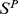 and 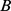, 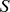 and 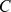, and 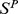 and 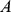. The former two mappings behave like the simple model (see above). The mapping between 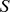 and 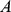 is a molecular code, because the molecular context of the system can be changed, such that the alternative system behavior is generated (Fig. 4D). The molecular context between 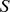 and 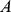 is either the set 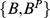, or alternatively 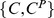. If we assume two concentration levels denoted by 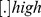 and 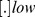 for high and low concentrations, respectively, we can identify the following codes: Applying the molecular context 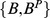 we get the mappings 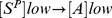, 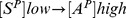, 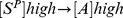, and 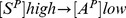, while the molecular context 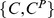 leads to the mappings 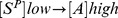, 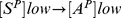, 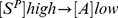, and 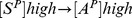. We simulated the system and applied both contexts 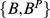 and 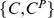. For the former context a change in 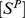 (x-axis) leads to a decrease in the 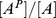-ratio (y-axis). Applying the alternative context 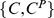 leads to the opposite behavior. Fig. 4 E illustrates these dependencies (for details of the underlying model see Text S3 ).

The extension of our static approach to a dynamic setting needs more strict definitions, such that the here shown properties are only a first step into this direction.

### Random Reaction Networks as Null Model

**Figure 5:**
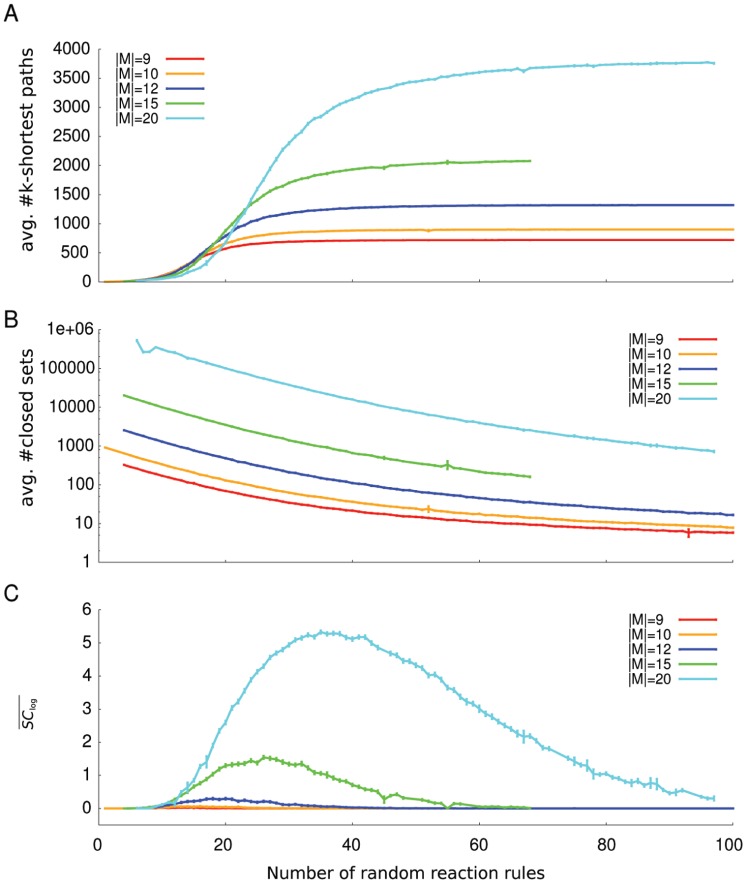
Structural properties of random reaction networks of different size and density. Panels A and B show two important network parameters for five different network sizes over the numbers of reaction rules. The data represents the average values of random replicates. Error bars indicate the standard error of the mean. Panel A shows the average number of paths in the network. Since we applied the path algorithm which only uses the k-shortest paths between each pair of molecular species the curve shows a sigmoidal behavior, which is saturated at the value 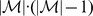, with 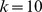. Panel B shows the average number of closed sets. With growing density the number of closed sets decreases. Panel C shows the distributions of the average number of code pairs (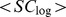). The semantic capacity follows a unimodal distribution indicating the existence of an optimal interval for the random generation of the BMC motif. If the number of paths is too low no mappings can be implemented because of the missing links between potential signs and meanings. Similarly, if the number of closed sets is too low no mappings can be implemented either.

**Figure 6:**
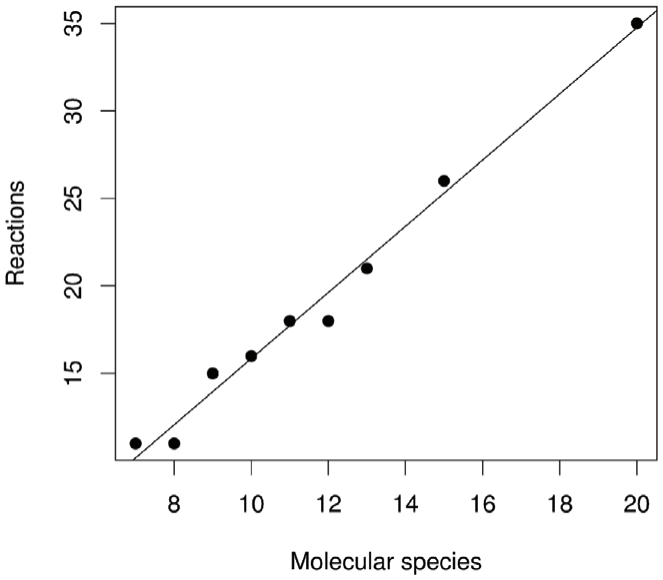
Maximal semantic capacity in random networks. Scatter plot showing the position of the maximal semantic capacity of the random reaction network data (cf. Fig. 5) in a 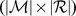-plot. The linear regression of the data shows that the maximal semantic capacity is reached if there are approximately two times more reactions in the system than molecular species: 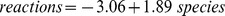.

To check whether the motif describing a BMC can be generated by chance we analyzed random reaction networks of different sizes and densities for their semantic capacity. The networks have been generated by random insertion of reaction rules in an empty network. Each random reaction rule is bimolecular, i.e. contains two reactants, and one product (see Text S1 for pseudocode). The analysis showed that the binary code motif can be generated in random networks (Fig. 5), i.e. contingent mappings can be generated randomly. For a fixed network size and varying densities the average semantic capacity shows a unimodal behavior, which suggests that there exist an optimal range of densities for each network size, leading to maximal semantic capacity. This optimal range shifts to higher densities with increasing size of the network (see Fig. 6). The optimal interval is bounded at lower densities by the low complexity of the network, there are not enough reactions to promote the insertion of molecular codes by chance. On higher densities the network is strongly connected, such that the subsets of the system are hardly closed, therefore it is also harder to implement codes by chance. The optimal interval coincides with two important network properties, i.e. the number of paths, and the number of closed sets. With increasing network density the number of paths grows, while the number of closed sets decreases. High semantic capacity can be found in networks with a high number of pathways and at the same time a high number of closed sets.

### Combustion Chemistries and the Martian Atmosphere Photochemistry Show no Semantic Capacity

We analyzed a number of chemical systems, i.e. combustion chemistries of hydrogen [47] (Dataset S6), methane [48] (Dataset S7), ethanol [49] (Dataset S8), dimethyl ether [50] (Dataset S9). The chemistries are intended to describe all significant processes that can occur in the combustion, i.e. burning, of the respective molecule. The original combustion chemistry data (provided in CHEMKIN format [43]) have been processed to obtain the reaction networks describing the respective chemistry. In the CHEMKIN files reactions are described at equilibrium with additional thermodynamic parameters. Taking these as basis we obtain reaction networks containing the directed reactions depending on the thermodynamic parameters.

The reaction networks cover different sizes (10–79 molecular species) and densities (38–752 reactions). The semantic analysis shows that none of these chemistries is able to realize molecular codes. We can now compare the results with our null model derived from the random reaction networks data (Table 5) to evaluate if both are consistent.

**Table 5:** Comparison of combustion chemistries and random networks (null model).

Combustion chemistry properties						Null model estimate		
	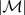	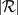	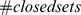	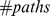	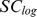	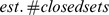 (SEM)	est. 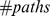	est. 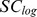
HYD	10	38	16	7.69  10^4^	0	39.84 (0.53)	878.15 (1.27)	0 (0.0)
MET	37	340	4,136	>10^6^	0	6,423.22 (209.75)	>1.33.10^4^	1.12 (0.08)
ETH	57	752	5,136	>10^6^	0	82,453.25 (9,545.96)	>3.19.10^4^	3.86 (0.36)
DME	79	708	8	>10^6^	0	n.a.	n.a.	n.a.

Values in brackets are the standard error of the mean (SEM). The analyzed combustion chemistries show no semantic capacity. This is supported by the analysis of random networks of the same size and density. The low number of codes found in these random networks can be explained by the noise of the random network generation. Number of random networks: 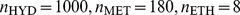, for DME the calculation was not feasible. All networks have been analyzed with the pathways based algorithm with 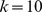. The number of paths in MET, ETH, DME has been estimated by counting paths with growing values of k. The maximal computable value gives a lower bound.

For the hydrogen chemistry the lack of code pairs can be explained by the small number of closed sets compared to the number of paths, such that the molecular species are “too connected” and the network is less structured. In the null model also no molecular codes can be identified. The estimated number of closed sets and paths, although differing from the original chemistry, are also marking that the respective random networks are not in the optimal interval.

In the methane combustion chemistry we see that there exist far more paths than closed sets, such that the network is to some extend “unstructured”. The according null model networks also contain a high number of paths, but also a higher number of closed sets. The algorithmic analysis shows that some of the generated null model networks can realize BMCs, such that the average logarithmic semantic capacity is 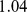. Nevertheless, we consider this also as a very low semantic capacity compared to, e.g. the gene translation chemistry. We also analyzed the atmosphere chemistry of Mars [51] (Dataset S5 ) to check whether other kinds of non-biological systems may contain codes. The model contains 32 molecular species, 104 reactions and 5512 closed sets. In particular, the network describes the reactions happening on the day side of mars. Therefore, light (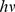) is modeled explicitly as inflow reaction 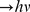. The day side martian photochemistry is not able to realize molecular codes. The comparison of the null model chemistries for ethanol, dimethyl ether, and the martian atmosphere chemistry were not feasible with our current algorithms, due to the large number of paths and closed sets in these networks.

### NTOP: An Artificial Chemistry Allowing for Molecular Coding

Recall that with increasing density random networks have a vanishing semantic capacity. In the following we show that even a dense network can have a relatively high semantic capacity. For this purpose we analyze an artificial chemistry with 16-species introduced by Banzhaf [52] called NTOP. For each species there is a 4-bit binary representation and the reaction rules are derived with respect to this representation, which is referred to as a structure-to-function mapping (see Ref. [52] for details and Dataset S10 for the network model).

The algorithmic analysis results in six code pairs (Text S4 ). Two properties of molecular codes that are of general importance also for biological molecular codes can be observed here. (1) A meaning can take the role of a sign in another code, and (2) molecular species can function as signs (or meanings) in different codes, i.e. they keep their role in different contexts (Fig. S1).

To test the robustness of the network’s semantic capacity, we replace 1, 2, 5, 10, 15, 200, and 1000 reaction rules randomly, respectively. In a randomly chosen reaction rule we replace the molecular species, while keeping the number of reactants and products the same. Thus, the type of the reaction stays the same, while the connections are changed. Increased randomization results in a decreased average semantic capacity (Fig. S2). Nevertheless in some cases the randomized network is capable to implement more code pairs. The general trend towards less code pairs can be explained by referring to the analysis of random reaction networks. Random reaction networks with the same number of species and reactions as NTOP show no semantic capacity (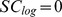). Thus the random variation of the NTOP chemistry drives the system towards the mean semantic capacity of random networks.

## Discussion

We have introduced a formal criterion for identifying molecular codes in reaction networks and a measure of the semantic capacity of a network, as the number of different code pairs the network can realize. Our notion of contingency, defined as the ability of systems to choose between different mappings, extends and operationalizes the notion of “independence” and “contingency” as discussed by Monod, Barbieri and others.

The structure of molecular codes allows to decompose them into binary molecular codes, which were studied here. Having a list of binary molecular codes it is possible to merge them into larger molecular codes, as has been demonstrated for the genetic code.

Applying the new concepts to different networks, our basic finding demonstrates that the semantic capacity of biological networks tends to be higher than the semantic capacity of the studied non-biological networks. Thus, an important step during the transition from non-life to life must have been the utilization of a chemistry that allows to implement molecular codes. In our opinion it is an open issue how that first coding chemistry has looked like. But, we have now a criterion that can guide us in what we have to look for. Following this line of thought it seems that biological systems “learned” by evolution to make use of chemistries with high semantic capacities by selecting the most appropriate mappings for their purpose. There exist at least three (not necessarily disjoint) evolutionary paths to select a unique mapping from the actual contingency: (1) *compartimentalization*, i.e. spatial separation of the two alternative mappings, (2) separation by *time* of execution, and (3) *fixation*, i.e. separation by deleting one of the alternative mappings. For the genetic code we could argue that at least two paths are used by cells to maintain the uniqueness of the mapping. Different codes are implemented in different species and compartments (compartimentalization) [22] and the genes for the alternative amino acyl tRNA synthetases are not present in the genome (fixation). Time separation can be understood as a regulated switch of mappings, e.g. in mitotic control where the presence of a protein called Cdc20 inhibits the Anaphase-Promoting Complex (APC) during the activated spindle assembly checkpoint (SAC), while in the context of the inactivated checkpoint, Cdc20 activates APC [53], [54].

Moreover, we can now precisely formulate another hypothesis, namely, that during the course of evolution the semantic capacity of the chemistry employed by the biological systems has a tendency to increase, by recruiting new chemistries, though the increase is not necessarily monotonous. One candidate mechanism is the invention and improvement of compositional adaptors, like proteins with exchangeable domains [55] or genes including their promoter- and coding-regions [9]. Note that also the appearance and evolution of neurons and cognitive systems is in line with the hypothesis of increasing semantic capacity.

The analysis of a network model implementing the genetic code showed that not only the codons can be signs, but also tRNA molecules could, in principle, be signs. Apparently, this potential code is not used by the cell. The biomolecular and evolutionary interpretation of this fact has to be left for future studies, because we have to make the notion of code *usage*, that is, the pragmatic aspect of biological information, more precise.

Furthermore, we have shown that DNA not only can function as a sign but also as a molecular context, as the study of gene regulatory networks revealed. The mechanisms in gene regulatory systems and the observation that such systems are highly flexible (i.e. the mapping between transcription factors and gene products can easily be changed) leads to the conclusion that the chemistry of GRNs possesses also a high semantic capacity. This may be the reason why it is the main regulatory subsystem of cells and often is used as typical representant of cellular information processing [56]. From a theoretical point of view it will be interesting to analyse more complex variants (several binding site, different types of transcription factors, transcription factor assembly) of the general GRN network for their influence on the semantic capacity. These extensions can introduce new codes by allowing for additional control and regulation of the system.

Phosphorylation cascades represent a class of biological systems that allow for molecular codes, but requires a quantitative analysis, i.e. the incorporation of concentrations. Thus our qualitative approach is not sufficient here. In the future the molecular code concept needs to be extended to the dynamic interpretation of a system. A molecular code then could be interpreted as a mapping between system states.

The analysis of random networks of different sizes and densities results in a better understanding of the basal rate of code occurrence. We can observe that the distribution of BMCs is unimodal, with high semantic capacity appearing only in sparsely connected random networks, in particular, where the number of second order reactions is approximately twice the number of molecular species. Interestingly, random networks with high semantic capacity show at the same time a high number of closed sets of species (which decreases with increasing network density) and a high number of paths (which increases with increasing network density). The null model estimates the semantic capacity of a reaction network that is generated completely by a random process. For biological and chemical systems this is obviously not true, because of physical constraints like mass conservation on the reactions.

The analysis of the artificial chemistry NTOP suggests that also in dense networks the semantic capacity can be high. We hypothesize that this was caused by the structure-to-function mapping applied in the definition of the chemistry.

There exist certain limitations on the kind of networks that should be analyzed with our approach. The definition of molecular codes requires that, to be applicable, the network model needs to contain all possible reactions among the molecular species. Network data widely available from databases like KEGG, Reactome, BioCyc, or Biomodels DB usually does not fulfill this criteria, yet. The networks found in these databases are becoming now rather complete with respect to the particular organism they belong to. However the network data is rather incomplete with respect to the underlying (bio-)chemistry. That is, with respect to the underlying chemistry many more alternative network species and reactions are possible, which cannot be found in those databases for several practical as well as conceptual reasons. It is the central innovation of our approach that for detecting a molecular code, we need to know the potential reaction network, which in general is not visible in the actual organism. It might sound a bit paradoxical that a network property depends on something that is not part of the network. In our case, however, the link to this “invisible” part is provided by physical laws and chemistry, which determine the alternative network species and reactions.

How to measure the semantic capacity of an actual biochemical system? We suggest a procedure consisting of three major steps: Step 1: Define the system to be studied and its chemistry, Step 2: Obtain the reaction network by physical experiments, Step 3: Compute all molecular codes of the network. In Step 1 we explicate the necessary assumptions: We define the chemical universe we will look at, i.e. the set of potential chemical species and the set of all possible reactions. Note that this depends on the time scale at which our system exists. At a longer timescale more reactions might have to be considered. Further assumptions can include constraints like temperature, pressure, pH, or energy consumption. In Step 2 we construct the reaction network using scientific physical experiments. Methods for this exist in a large variety in Chemistry and the Life Sciences. Note that with proper assumptions (Step 1) we approach with increasing number of experiments a single unique network. In other words, there is a single “true” network, which is defined by the scientific procedure and the assumptions made in Step 1. At least in principle, we can obtain this network with arbitrary precision, provided arbitrary but finite experimental resources. As an open problem remains the question how a measurement error on the network level propagates to the estimation of the semantic capacity. Step 3 is purely formal and in principle deterministic. Practically, however, for large and complex networks (e.g., networks with more than 1000 species) the run time of our deterministic algorithms described here is too long and thus efficient heuristics have to be developed for these networks in the future.

In summary, we conclude that our approach provides a new way to analyze aspects of the information processing capabilities of molecular systems, which might contribute to the understanding of biological information in the context of the origin and evolution of life, cellular signaling, or synthetic molecular computing systems.

## Supporting Information

Figure S1
**Relation among the code pairs in the NTOP chemistry.** Graph illustrating the six code pairs found in the NTOP chemistry. The nodes refer to the closed sets containing the signs and meanings in each individual code pair. The six code pairs are distinguished by color. The graph clearly shows that signs and meanings can be reused in different codes and also change their role, i.e. meanings can be sign in another code, e.g. 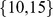.(EPS)

Figure S2 **Effect of network randomization on the semantic capacity.** The boxplots shows the relation between semantic capacity and increasing randomization for the artificial chemistry NTOP. With increasing randomizing the semantic capacity decreases on average. Nevertheless, weak to medium randomization, i.e. only parts of the network are randomly rearranged, can also lead to higher semantic capacity, while after very high randomization this effect does not appear. The boxplots show the distribution of the semantic capacity after 100 independent randomizations of the chemistry by replacing a fixed number of reaction rules.
(EPS)

Table S1 **List of all analyzed systems stating their size, density, semantic capacity, the reference of the system, and the method used for analysis.**
(PDF)

Table S2 **Reaction network formulation of a gene translation system with amino-acyl-tRNA-synthetases.**
(PDF)

Text S1 **Pseudocode of the closure-base code identifying algorithms, the pathway-based code identifying algorithm and the random network generation algorithm.**
(PDF)

Text S2 **List of Molecular Codes that can be identified in the merge of the 17 known genetic codes. For the network see Dataset S3.**
(PDF)

Text S3 **Mathematical model of the phosphorylation cascade shown in** **Figure 4C** **.**
(PDF)

Text S4 **List of all binary molecular codes (including duplicates) identified in the NTOP chemistry.**
(PDF)

Dataset S1 **Network model of** **Figure 1A** **.**
(TXT)

Dataset S2 **Network model of** **Figure 1C** **.**
(TXT)

Dataset S3 **Network model the merge of the 17 known genetic codes as listed at NCBI.**
(TXT)

Dataset S4 **Network model of a 2×2 subnetwork of the gene translation chemistry including synthetases.**
(TXT)

Dataset S5 **Network model of the Martian atmosphere photochemistry.**
(TXT)

Dataset S6 **Network model of hydrogen combustion.**
(TXT)

Dataset S7 **Network model of methane combustion.**
(TXT)

Dataset S8 **Network model of ethanol combustion.**
(TXT)

Dataset S9 **Network model of di methyl ether combustion.**
(TXT)

Dataset S10 **Network model of the artificial chemistry NTOP.**
(TXT)

## References

[1] Shannon CE (1948) A mathematical theory of communication. The Bell Systems Technical Journal 27: 379–423, 623–656.

[2] Tkačik G, Walczak AM (2011) Information transmission in genetic regulatory networks: A review. J Phys Condens Matter 23: 153102. 21460423 10.1088/0953-8984/23/15/153102

[3] Mehta P, Goyal S, Long T, Bassler BL, Wingreen NS (2009) Information processing and signal integration in bacterial quorum sensing. Mol Syst Biol 5: 325. 19920810 10.1038/msb.2009.79 PMC2795473

[4] Lenaerts T, Ferkinghoff-Borg J, Stricher F, Serrano L, Schymkowitz JWH, et al (2008) Quantifying information transfer by protein domains: Analysis of the Fyn SH2 domain structure. BMC Struct Biol 8: 43. 18842137 10.1186/1472-6807-8-43 PMC2585567

[5] Waltermann C, Klipp E (2011) Information theory based approaches to cellular signaling. Biochim Biophys Acta General Subjects 1810: 924–932. 10.1016/j.bbagen.2011.07.009 21798319

[6] Monod J (1971) *Chance and necessity*. Alfred Knopf, New York/NY. (Originally published 1970).

[7] Küppers BO (1990) *Information and the origin of life*. MIT Press, Cambridge/MA. (Originally published 1986).

[8] Tsuda S, Artmann S, Zauner KP (2009) The Phi-Bot. In Adamatzky A, Komosinski M, eds., Artificial Life models in hardware. Springer, Dordrecht, 213–232.

[9] Barbieri M (2008) Biosemiotics: a new understanding of life. Naturwissenschaften 95: 577–599. 18365164 10.1007/s00114-008-0368-x

[10] Koonin EV, Novozhilov AS (2009) Origin and evolution of the genetic code: the universal enigma. IUBMB Life 61: 99–111. 19117371 10.1002/iub.146 PMC3293468

[11] Pattee HH (2008) Physical and functional conditions for symbols, codes, and languages. Biosemiotics 1: 147–168.

[12] Görlich D, Dittrich P (2011) Identifying molecular organic codes in reaction networks. In Kampis G, Karsai I, Szathmáry E, eds., Advances in Artificial Life. Darwin Meets von Neumann, vol. 5777 of Lecture Notes in Computer Science. Springer Berlin/Heidelberg, 305–312.

[13] Fontana W, Buss L (1994) The arrival of the fittest: Toward a theory of biological organization. Bull Math Bio 56: 1–64.

[14] Speroni di Fenizio P, Dittrich P, Ziegler J, Banzhaf W (2000) Towards a theory of organizations. In Lange H, et al. (Eds.) German Workshop on Artificial Life (GWAL 2000), in print. Bayreuth, 5.-7. April, 2000, available online: http: //di.ttri.ch/p/SDZB2001gwal.pdf.

[15] Tlusty T (2008) Casting polymer nets to optimize noisy molecular codes. Proc Natl Acad Sci U S A 105: 8238–8243. 18550822 10.1073/pnas.0710274105 PMC2448821

[16] Tlusty T (2008) Rate-distortion scenario for the emergence and evolution of noisy molecular codes. Phys Rev Lett 100: 048101. 18352335 10.1103/PhysRevLett.100.048101

[17] Martins EQV, Pascoal MMB (2003) A new implementation of yen’s ranking loopless paths algorithm. 4OR: A Quarterly Journal of Operations Research 1: 121–133.

[18] Crick FH, Barnett L, Brenner S, Watts-Tobin RJ (1961) General nature of the genetic code for proteins. Nature 192: 1227–1232. 13882203 10.1038/1921227a0

[19] De Beule J, Hovig E, Benson M (2011) Introducing dynamics into the field of biosemiotics. Biosemiotics 4: 5–24.

[20] Osawa S, Jukes TH, Watanabe K, Muto A (1992) Recent evidence for evolution of the genetic code. Microbiol Rev 56: 229–264. 1579111 10.1128/mr.56.1.229-264.1992 PMC372862

[21] Jukes TH, Osawa S (1993) Evolutionary changes in the genetic code. Comp Biochem Physiol B 106: 489–494. 8281749 10.1016/0305-0491(93)90122-l

[22] Elzanowski A, Ostell J (2010) The genetic code. Available: http: //www.ncbi.nlm.nih.gov/Taxonomy/Utils/wprintgc.cgi, version 3.9, July 07, 2010. Accessed 2011 February 20.

[23] Liu CC, Schultz PG (2010) Adding new chemistries to the genetic code. Annu Rev Biochem 79: 413–444. 20307192 10.1146/annurev.biochem.052308.105824

[24] Clark-Walker GD, Weiller GF (1994) The structure of the small mitochondrial DNA of Kluyveromyces thermotolerans is likely to reflect the ancestral gene order in fungi. J Mol Evol 38: 593–601. 8083884 10.1007/BF00175879

[25] Himeno H, Masaki H, Kawai T, Ohta T, Kumagai I, et al (1987) Unusual genetic codes and a novel gene structure for tRNA(AGYSer) in starfish mitochondrial DNA. Gene 56: 219–230. 3678836 10.1016/0378-1119(87)90139-9

[26] Jacobs HT, Elliott DJ, Math VB, Farquharson A (1988) Nucleotide sequence and gene organization of sea urchin mitochondrial DNA. J Mol Biol 202: 185–217. 3172215 10.1016/0022-2836(88)90452-4

[27] Batuecas B, Garesse R, Calleja M, Valverde JR, Marco R (1988) Genome organization of Artemia mitochondrial DNA. Nucleic Acids Res 16: 6515–6529. 3135541 10.1093/nar/16.14.6515 PMC338311

[28] Osawa S, Ohama T, Jukes TH, Watanabe K (1989) Evolution of the mitochondrial genetic code. I. Origin of AGR serine and stop codons in metazoan mitochondria. J Mol Evol 29: 202–207. 2506356 10.1007/BF02100203

[29] Garey JR, Wolstenholme DR (1989) Platyhelminth mitochondrial DNA: Evidence for early evolutionary origin of a tRNA(serAGN) that contains a dihydrouridine arm replacement loop, and of serine-specifying AGA and AGG codons. J Mol Evol 28: 374–387. 2545889 10.1007/BF02603072

[30] Ohama T, Osawa S, Watanabe K, Jukes TH (1990) Evolution of the mitochondrial genetic code. IV. AAA as an asparagine codon in some animal mitochondria. J Mol Evol 30: 329–332. 2111847 10.1007/BF02101887

[31] Hoffmann RJ, Boore JL, Brown WM (1992) A novel mitochondrial genome organization for the blue mussel, Mytilus edulis. Genetics 131: 397–412. 1386586 10.1093/genetics/131.2.397 PMC1205014

[32] Durrheim GA, Corfield VA, Harley EH, Ricketts MH (1993) Nucleotide sequence of cytochrome oxidase (subunit III) from the mitochondrion of the tunicate Pyura stolonifera: evidence that AGR encodes glycine. Nucleic Acids Res 21: 3587–3588. 8393993 10.1093/nar/21.15.3587 PMC331473

[33] Boore JL, Brown WM (1994) Complete DNA sequence of the mitochondrial genome of the black chiton, Katharina tunicata. Genetics 138: 423–443. 7828825 10.1093/genetics/138.2.423 PMC1206160

[34] Kondow A, Suzuki T, Yokobori S, Ueda T, Watanabe K (1999) An extra tRNAGly(U*CU) found in ascidian mitochondria responsible for decoding non-universal codons AGA/AGG as glycine. Nucleic Acids Res 27: 2554–9. 10352185 10.1093/nar/27.12.2554 PMC148460

[35] Telford MJ, Herniou EA, Russell RB, Littlewood DT (2000) Changes in mitochondrial genetic codes as phylogenetic characters: two examples from the flatworms. Proc Natl Acad Sci U S A 97: 11359–11364. 11027335 10.1073/pnas.97.21.11359 PMC17205

[36] Yokobori S, Watanabe Y, Oshima T (2003) Mitochondrial genome of Ciona savignyi (Urochordata, Ascidiacea, Enterogona): Comparison of gene arrangement and tRNA genes with Halocynthia roretzi mitochondrial genome. J Mol Evol 57: 574–587. 14738316 10.1007/s00239-003-2511-9

[37] Nedelcu AM, Lee RW, Lemieux C, Gray MW, Burger G (2000) The complete mitochondrial DNA sequence of Scenedesmus obliquus reflects an intermediate stage in the evolution of the green algal mitochondrial genome. Genome Res 10: 819–831. 10854413 10.1101/gr.10.6.819 PMC310893

[38] Hayashi-Ishimaru Y, Ohama T, Kawatsu Y, Nakamura K, Osawa S (1996) UAG is a sense codon in several chlorophycean mitochondria. Curr Genet 30: 29–33. 8662206 10.1007/s002940050096

[39] Laforest MJ, Roewer I, Lang BF (1997) Mitochondrial tRNAs in the lower fungus Spizellomyces punctatus: tRNA editing and UAG ‘stop’ codons recognized as leucine. Nucleic Acids Res 25: 626–632. 9016605 10.1093/nar/25.3.626 PMC146481

[40] Schneider SU, Leible MB, Yang XP (1989) Strong homology between the small subunit of ribulose-1,5-bisphosphate carboxylase/oxygenase of two species of Acetabularia and the occurrence of unusual codon usage. Mol Gen Genet 218: 445–452. 2573818 10.1007/BF00332408

[41] Schneider SU, de Groot EJ (1991) Sequences of two rbcS cDNA clones of Batophora oerstedii: structural and evolutionary considerations. Curr Genet 20: 173–175. 1934113 10.1007/BF00312782

[42] Liang A, Heckmann K (1993) Blepharisma uses UAA as a termination codon. Naturwissenschaften 80: 225–226. 7685500 10.1007/BF01175738

[43] Keeling PJ, Doolittle WF (1996) A non-canonical genetic code in an early diverging eukaryotic lineage. EMBO J 15: 2285–2290. 8641293 PMC450153

[44] Kaufmann A, Knop M (2011) Genomic promoter replacement cassettes to alter gene expression in the yeast saccharomyces cerevisiae. Methods Mol Biol 765: 275–294. 21815098 10.1007/978-1-61779-197-0_16

[45] Brakhage AA, Schroeckh V (2011) Fungal secondary metabolites - strategies to activate silent gene clusters. Fungal Genet Biol 48: 15–22. 20433937 10.1016/j.fgb.2010.04.004

[46] Krauss G (2008) Biochemistry of Signal Transduction and Regulation. Wiley-VCH, Weinheim, 4 edn.

[47] Conaire MO, Curran HJ, Simmie JM, Pitz WJ, Westbrook C (2004) A comprehensive modeling study of hydrogen oxidation. Int J Chem Kinet 36: 603–622.

[48] Hughes KJ, Turanyi T, Clague AR, Pilling MJ (2001) Development and Testing of a comprehensive chemical mechanism for the oxidation of methane. Int J Chem Kinet 33: 513–538.

[49] Marinov NM (1999) A detailed chemical kinetic model for high temperature ethanol oxidation. Int J Chem Kinet 31: 183–220.

[50] Kaiser E, Wallington T, Hurley MD, Platz J, Curran HJ, et al (2000) Experimental and modeling study of premixed atmospheric-pressure dimethyl ether-air flames. J Phys Chem 104: 8194–8206.

[51] Nair H, Allen M, Anbar AD, Yung YL (1994) A photochemical model of the martian atmosphere. Icarus 111: 124–150. 11539176 10.1006/icar.1994.1137

[52] Banzhaf W (1993) Self-replicating sequences of binary numbers. Comput Math Appl 26: 1–8.

[53] Musacchio A, Salomon ED (2007) The spindle-assembly checkpoint in space and time. Nat Rev Mol Cell Bio 8: 379–393. 17426725 10.1038/nrm2163

[54] Ibrahim B, Diekmann S, Schmitt E, Dittrich P (2008) In-silico modeling of the mitotic spindle assembly checkpoint. PLoS One 3(2): e1555. 18253502 10.1371/journal.pone.0001555 PMC2215771

[55] Bornberg-Bauer E, Huylmans AK, Sikosek T (2010) How do new proteins arise? Curr Opin Struct Biol 20: 390–396. 20347587 10.1016/j.sbi.2010.02.005

[56] Tyson JJ, Novak B (2010) Functional motifs in biochemical reaction networks. Annu Rev Phys Chem 61: 219–240. 20055671 10.1146/annurev.physchem.012809.103457 PMC3773234

