# Supplementary material for: Molecular Codes in Biological and Chemical Reaction Networks
Source: PLoS One. 2013 Jan 23;8(1):e54694. doi: 10.1371/journal.pone.0054694 (PMC3553058; doi:10.1371/journal.pone.0054694)
Supplement: Table S1 — List of all analyzed systems stating their size, density, semantic capacity, the reference of the system, and the method used for analysis. (PDF) [file pone.0054694.s003.pdf]

List of all analyzed systems stating their size, density, semantic capacity, the reference of the system, and the method used for analysis.

Abbrev	#species	#reactions	#closed sets	#paths	SC_{log}	Method	Reference	Description
FIG1A	8	4	161	12	1	c & p	this study	Network from Figure 1A.
FIG1C	6	4	41	16	1.58	c & p	this study	Network from Figure 1C.
GCMERGE	234	85	n.a.	170	4.09	p	this study	Network reconstructed from the genetic codes reported at [1]
GCFULL	1364	1280	n.a.	n.a	18.55	t	this study	Theoretical estimate of SC_{log} of a network, based on GCMERGE, generated by inserting all possible mappings between codons and amino acids
GCFULLSYNTSMALL	16	8	n.a.	200	2.32	p	this study	Network with two codons, two amino acids, and synthetases.
GCFULLSYNT	2,728	2,560	n.a.	n.a.	20.55	t	this study	Theoretical evaluation of a reaction network containing all possible mappings between the 64 codons and 20 amino acids with synthetases
MARS	32	104	5,512	$> 10^6$	0	c	[6]	Chemical processes occurring in the Martian atmosphere during the daylight phase
HYD	10	38	16	$7.69 \cdot 10^4$	0	c	[2]	Combustion chemistry of hydrogen
MET	37	340	4,136	$> 10^6$	0	c	[3]	Combustion chemistry of methane
ETH	57	752	5,136	n.a.	0	c	[4]	Combustion chemistry of ethanol
DME	79	708	8	$> 10^6$	0	c	[5]	Combustion chemistry of dimethyl ether
NTOP	16	207	244	$474, 218$	2.81	c & p	[7]	Artificial chemistry based on binary strings operations
R_NTOP	16	207	18.11 (sem=0.23)	n.a.	0 (sem=0)	c	this study	Average on 1000 random networks of the same size and density as the NTOP network.
RANDOM	varies	varies	varies	varies	varies	c & p	this study	Analysis of different random networks.

Abbrev.: c - closure based algorithm, p - pathway-based algorithm, t - theoretical analysis, SC_{log} - logarithmized semantic capacity, n.a. - not available, sem - standard error of the mean

*: determined with $k = 10000$.

References

[1] Elzanowski, A & Ostell, J. (2010) The genetic code. http://www.ncbi.nlm.nih.gov/Taxonomy/Utils/wprintgc.cgi, version 3.9, July 07, 2010, retrieved: Feb 20, 2011.

[2] Conaire, M. O, Curran, H. J, Simmie, J. M, Pitz, W. J, & Westbrook, C. (2004) *Int J Chem Kinet* **36**, 603–622.

[3] Hughes K. J., Turanyi T., Clague A. R. , Pilling M. J. (2001) Development and Testing of a comprehensive chemical mechanism for the oxidation of methane *Int J Chem Kinet* 33:513–538.

[4] Marinov, N. M. (1999) *Int. J. Chem. Kinet.* **31**, 183–220.

[5] Kaiser, E, Wallington, T, Hurley, M. D, Platz, J, Curran, H. J, Pitz, W. J, & Westbrook, C. K. (2000) *Journal of Physical Chemistry* **104**, 8194–8206.

[6] Nair, H, Allen, M, Anbar, A. D, & Yung, Y. L. (1994) *Icarus* **111**, 124–150.

[7] Banzhaf, W. (1993) *Comput. Math. Appl.* **26**, 1–8.
