# Supplementary material for: Molecular Codes in Biological and Chemical Reaction Networks
Source: PLoS One. 2013 Jan 23;8(1):e54694. doi: 10.1371/journal.pone.0054694 (PMC3553058; doi:10.1371/journal.pone.0054694)
Supplement: Table S2 — Reaction network formulation of a gene translation system with amino-acyl-tRNA-synthetases. (PDF) [file pone.0054694.s004.pdf]

Reaction network formulation of a gene translation system with amino-acyl-tRNA-synthetases

Eq.	Definition	Description
1	$\mathcal{M}_{GC} = Codons \cup AA^{free} \cup AA^{prot} \cup aaRS \cup tRNA^{free} \cup tRNA^{loaded}$	Definition of the molecular species in the network
2	$Codons = \{\mathrm{A,C,G,T}\}^3 = \{\mathrm{AAA}, \mathrm{AAC}, \ldots, \mathrm{TTT}\}$	Set representing the 64 codons of the genetic code
3	$AA^{free} = \{\mathrm{Ala}^{\mathrm{free}}, \mathrm{Arg}^{\mathrm{free}}, \mathrm{Asp}^{\mathrm{free}}, \ldots, \mathrm{Try}^{\mathrm{free}}\}$	Amino acids that are not used in a protein
4	$AA^{prot} = \{\mathrm{Ala}^{\mathrm{prot}}, \mathrm{Arg}^{\mathrm{prot}}, \mathrm{Asp}^{\mathrm{prot}}, \ldots, \mathrm{Try}^{\mathrm{prot}}\}$	Amino acids that have been used in a protein during gene translation
5	$tRNA^{free} = \{\mathrm{tRNA}_n \| n \in Codons\}$	Unloaded tRNAs specific for codon n
6	$tRNA^{loaded} = \{\mathrm{tRNA}_{n,a} \| n \in Codons, a \in AA_{free}\}$	tRNAs specific for codon n that have been loaded with amino acid a
7	$aaRS = \{\mathrm{Syn}_{n,a} \| n \in Codons, a \in AA_{free}\}$	Amino-acyl-tRNA-synthetases that are specific for amino acid a and codon n
8	$\mathcal{R}_{GC} = \{\mathrm{tRNA}_n + a + \mathrm{Syn}_{n,a} \rightarrow tRNA_{a,n} + Syn_{n,a} \mid n \in Codons, a \in AA^{free}\} \cup$	Loading of the tRNA by suitable synthetasis
9	$\{n + \mathrm{tRNA}_{a,n} \rightarrow n + \mathrm{tRNA}_n + a \mid n \in Codons, a \in AA^{prot}\}$	Translation step, i.e., the incorporation of an amino acid into a growing protein
