# Supplementary material for: Molecular Codes in Biological and Chemical Reaction Networks
Source: PLoS One. 2013 Jan 23;8(1):e54694. doi: 10.1371/journal.pone.0054694 (PMC3553058; doi:10.1371/journal.pone.0054694)
Supplement: Text S1 — Pseudocode of the closure-base code identifying algorithms, the pathway-based code identifying algorithm and the random network generation algorithm. (PDF) [file pone.0054694.s005.pdf]

Pseudocode: Closure-based Algorithm

Algorithm 1 closureCodeFinder(N) - Closure Based Code Finder

Input: A reaction network $N = \langle \mathcal{M}, \mathcal{R} \rangle$ with molecular species $\mathcal{M}$ and reactions $\mathcal{R}$.
Output: A list of code pairs consisting of a set of signs, meanings and two codemakers.

clos ← allClosedSets($\mathcal{M}$)
single_mol_clos ← $\emptyset$
for all $m \in \mathcal{M}$ **do**
  $single\_mol\_clos \leftarrow single\_mol\_clos \cup \{generateClosure(m)\}$
end for
for all $S_1, S_2, M_1, M_2 \in single\_mol\_clos$ **do**
  for all $C, C' \in clos$ **do**
    if $M_1 \subseteq generateClosure(S_1 \cup C) \wedge M_2 \nsubseteq generateClosure(S_1 \cup C) \wedge$
    $M_2 \subseteq generateClosure(S_2 \cup C) \wedge M_1 \nsubseteq generateClosure(S_2 \cup C) \wedge$
    $M_2 \subseteq generateClosure(S_1 \cup C') \wedge M_1 \nsubseteq generateClosure(S_1 \cup C') \wedge$
    $M_1 \subseteq generateClosure(S_2 \cup C') \wedge M_2 \nsubseteq generateClosure(S_2 \cup C') \wedge$ **then**
      print $(S_1, S_2, M_1, M_2, C, C')$ as result
    end if
  end for
end for

Algorithm 2 allClosedSets(A) - Finds all closed sets of a set A

Input: A set A of molecular species from network N.
Output: A set $result$ containing all closed sets of A with respect to network N.

$largest \leftarrow generateClosure(A)$
$smallest \leftarrow generateClosure(\{\emptyset\})$
$clos\_to\_check \leftarrow smallest$
while $size(clos\_to\_check) \geq 0$ **do**
  $c \leftarrow clos\_to\_check.getFirst()$ {returns the first element from closToCheck}
  $usable \leftarrow largest - (c \cap largest)$
  $closures\_found \leftarrow findClosAbove(c, usable)$ {Finds all closures above c that contains a “usable” molecule}
  $closures\_to\_check \leftarrow closures\_to\_check - -\{c\}$
  $result \leftarrow result \cup \{c\}$
  $closure\_to\_check \leftarrow closure\_to\_check \cup (closures\_found - (closure\_found \cap result))$
end while
return result

Algorithm 3 generateClosure(A) - Generates the closure of an input set

Input: An input set $A \subseteq \mathcal{M}$.
Output: A set $B \subseteq \mathcal{M}$ representing the closed set induced by A.

repeat
  $B \leftarrow A$
  $A \leftarrow sqr(B) \cup B$
until $B == A$
return B

Algorithm 4 sqr(A) - Finds all molecular species that can be directly produced by a reaction

Input: An input set $A \subseteq \mathcal{M}$.
Output: Returns a set $result \subseteq \mathcal{M}$ that can be produced directly by reactions among molecules from A.

```
for all rea ∈ R do
  reactants ← getReactants(rea)
  if reactants ⊆ A then
    result ← result ∪ getProducts(rea)
  end if
end for
return result
```

Pseudocode: Path-based Algorithm

Algorithm 5 pathCodeFinder(N) - finds all molecular codes by path analysis of the network, based on a K-shortest path algorithm

Input: A reaction network $N = \langle \mathcal{M}, \mathcal{R} \rangle$ with molecular species $\mathcal{M}$ and reactions $\mathcal{R}$.
A natural number k defining the number of shortest path took into the analysis.
Output: A list of all code pairs the network can realize

```
for all s ∈ M do
  for all t ∈ M do
    paths^st ← getKShortestPaths(s, t, k)
  end for
end for
for all s, t, u, v ∈ M do
  for all p^st ∈ paths^st do
    for all p^uv ∈ paths^uv do
      for all p^sv ∈ paths^sv do
        for all p^ut ∈ paths^ut do
          c ← getContext(p^st) ∪ getContext(p^uv)
          c2 ← getContext(p^sv) ∪ getContext(p^ut)
          clos_s_c ← generateClosure({s} ∪ c)
          clos_u_c ← generateClosure({u} ∪ c)
          clos_s_c2 ← generateClosure({s} ∪ c2)
          clos_u_c2 ← generateClosure({u} ∪ c2)
          if t ∈ clos_s_c ∧ v ∉ clos_s_c ∧ t ∉ clos_u_c ∧ v ∈ clos_u_c ∧ t ∉ clos_s_c2 ∧ v ∈ clos_s_c2 ∧ t ∈ clos_u_c2 ∧ v ∉
          clos_u_c2∧ then
            print (s,t,u,v,c,c2) to the code pair list
          end if
        end for
      end for
    end for
  end for
end for
```

Algorithm 6 getContext(p) - gets the context of a s-t-path

Input: A reaction path $p = (r_1, r_2, \ldots, r_n)$ from s to t.
Output: A set of molecular species *context* which are the molecular context of p.

```
context ← ∅
for all r_i ∈ p do
  reactants ← getReactants(r_i)
  startset ← context ∪ {s}
  clos ← generateClosure(startset)
  new_context ← (reactants \ clos)
  context ← context ∪ new_context
end for
return context
```

Pseudocode: Random Network Generation

Algorithm 7 generateRandomNetwork()

Input: The size n of the network and the number of reactions m
Output: A random reaction network $N_{rand} = \langle \mathcal{M}, \mathcal{R} \rangle$, with $|\mathcal{M}| = n$ and $|\mathcal{R}| = m$.

```
M ← M ∪ {1} ∪ {2} ∪ ··· ∪ {n}
R ← ∅
for i in 1 to m do
    s1 ← random(1, n)
    s2 ← random(1, n)
    s3 ← random(1, n)
    R ← R ∪ {s1 + s2 → s3}
end for
return N
```

Algorithm 8 random()

Input: range (a,b).
Output: An integer value.

```
return Draw a random integer between a and b.
```
