# Supplementary material for: Molecular Codes in Biological and Chemical Reaction Networks
Source: PLoS One. 2013 Jan 23;8(1):e54694. doi: 10.1371/journal.pone.0054694 (PMC3553058; doi:10.1371/journal.pone.0054694)
Supplement: Text S2 — List of Molecular Codes that can be identified in the merge of the 17 known genetic codes. For the network see Dataset S3. (PDF) [file pone.0054694.s006.pdf]

List of Molecular Codes that can be identified in the merge of the 17 known genetic codes. For the underlying network see Dataset S3.

```
1.0
------Code-----
Sign 1  = [CTT, ]
Sign 2  = [CTG, ]
Meaning 1  = [L, ]
Meaning 2  = [T, ]
Codemaker  = [tRNACTTL, tRNACTGT, ]
alt. Codemaker  = [tRNACTTT, tRNACTGL, ]
S1->M1 = [83, 251, 40]
S2->M1 = [198, 258, 40]
S1->M2 = [83, 310, 51]
S2->M2 = [198, 234, 51]
-----END OF CODE ------


1.0
------Code-----
Sign 1  = [CTT, ]
Sign 2  = [CTA, ]
Meaning 1  = [L, ]
Meaning 2  = [T, ]
Codemaker  = [tRNACTAT, tRNACTTL, ]
alt. Codemaker  = [tRNACTAL, tRNACTTT, ]
S1->M1 = [83, 251, 40]
S2->M1 = [199, 278, 40]
S1->M2 = [83, 310, 51]
S2->M2 = [199, 281, 51]
-----END OF CODE ------


1.0
------Code-----
Sign 1  = [CTT, ]
Sign 2  = [CTC, ]
Meaning 1  = [L, ]
Meaning 2  = [T, ]
Codemaker  = [tRNACTTL, tRNACTCT, ]
alt. Codemaker  = [tRNACTCL, tRNACTTT, ]
S1->M1 = [83, 251, 40]
S2->M1 = [201, 238, 40]
S1->M2 = [83, 310, 51]
S2->M2 = [201, 248, 51]
```

```
-----END OF CODE ------


1.0
------Code-----
Sign 1  = [AGG, ]
Sign 2  = [AGA, ]
Meaning 1  = [G, ]
Meaning 2  = [STOP, ]
Codemaker  = [tRNAAGGG, tRNAAGASTOP, ]
alt. Codemaker  = [tRNAAGGSTOP, tRNAAGAG, ]
S1->M1 = [106, 241, 34]
S2->M1 = [109, 293, 34]
S1->M2 = [106, 318, 44]
S2->M2 = [109, 242, 44]
-----END OF CODE ------


1.0
------Code-----
Sign 1  = [AGG, ]
Sign 2  = [AGA, ]
Meaning 1  = [G, ]
Meaning 2  = [S, ]
Codemaker  = [tRNAAGGG, tRNAAGAS, ]
alt. Codemaker  = [tRNAAGGS, tRNAAGAG, ]
S1->M1 = [106, 241, 34]
S2->M1 = [109, 293, 34]
S1->M2 = [106, 304, 57]
S2->M2 = [109, 279, 57]
-----END OF CODE ------


1.0
------Code-----
Sign 1  = [AGG, ]
Sign 2  = [AGA, ]
Meaning 1  = [G, ]
Meaning 2  = [R, ]
Codemaker  = [tRNAAGGG, tRNAAGAR, ]
alt. Codemaker  = [tRNAAGGR, tRNAAGAG, ]
S1->M1 = [106, 241, 34]
S2->M1 = [109, 293, 34]
S1->M2 = [106, 302, 58]
S2->M2 = [109, 277, 58]
-----END OF CODE ------
```

```
1.0
------Code-----
Sign 1  = [AGG, ]
Sign 2  = [AGA, ]
Meaning 1  = [STOP, ]
Meaning 2  = [S, ]
Codemaker  = [tRNAAGGSTOP, tRNAAGAS, ]
alt. Codemaker  = [tRNAAGGS, tRNAAGASTOP, ]
S1->M1 = [106, 318, 44]
S2->M1 = [109, 242, 44]
S1->M2 = [106, 304, 57]
S2->M2 = [109, 279, 57]
-----END OF CODE ------
```

```
1.0
------Code-----
Sign 1  = [AGG, ]
Sign 2  = [AGA, ]
Meaning 1  = [STOP, ]
Meaning 2  = [R, ]
Codemaker  = [tRNAAGGSTOP, tRNAAGAR, ]
alt. Codemaker  = [tRNAAGGR, tRNAAGASTOP, ]
S1->M1 = [106, 318, 44]
S2->M1 = [109, 242, 44]
S1->M2 = [106, 302, 58]
S2->M2 = [109, 277, 58]
-----END OF CODE ------
```

```
1.0
------Code-----
Sign 1  = [AGG, ]
Sign 2  = [AGA, ]
Meaning 1  = [S, ]
Meaning 2  = [R, ]
Codemaker  = [tRNAAGGS, tRNAAGAR, ]
alt. Codemaker  = [tRNAAGGR, tRNAAGAS, ]
S1->M1 = [106, 304, 57]
S2->M1 = [109, 279, 57]
S1->M2 = [106, 302, 58]
S2->M2 = [109, 277, 58]
-----END OF CODE ------
```

```
1.0
------Code-----
Sign 1  = [AGG, ]
Sign 2  = [TCA, ]
Meaning 1  = [STOP, ]
Meaning 2  = [S, ]
Codemaker  = [tRNAAGGSTOP, tRNATCAS, ]
alt. Codemaker  = [tRNAAGGS, tRNATCASTOP, ]
S1->M1 = [106, 318, 44]
S2->M1 = [141, 274, 44]
S1->M2 = [106, 304, 57]
S2->M2 = [141, 272, 57]
-----END OF CODE ------


1.0
------Code-----
Sign 1  = [AGA, ]
Sign 2  = [TCA, ]
Meaning 1  = [STOP, ]
Meaning 2  = [S, ]
Codemaker  = [tRNAAGASTOP, tRNATCAS, ]
alt. Codemaker  = [tRNAAGAS, tRNATCASTOP, ]
S1->M1 = [109, 242, 44]
S2->M1 = [141, 274, 44]
S1->M2 = [109, 279, 57]
S2->M2 = [141, 272, 57]
-----END OF CODE ------


1.0
------Code-----
Sign 1  = [TTA, ]
Sign 2  = [TAG, ]
Meaning 1  = [L, ]
Meaning 2  = [STOP, ]
Codemaker  = [tRNATTAL, tRNATAGSTOP, ]
alt. Codemaker  = [tRNATTASTOP, tRNATAGL, ]
S1->M1 = [133, 316, 40]
S2->M1 = [223, 305, 40]
S1->M2 = [133, 303, 44]
S2->M2 = [223, 236, 44]
-----END OF CODE ------
```

```
1.0
------Code-----
Sign 1  = [CTG, ]
Sign 2  = [CTA, ]
Meaning 1  = [L, ]
Meaning 2  = [T, ]
Codemaker  = [tRNACTAT, tRNACTGL, ]
alt. Codemaker  = [tRNACTAL, tRNACTGT, ]
S1->M1 = [198, 258, 40]
S2->M1 = [199, 278, 40]
S1->M2 = [198, 234, 51]
S2->M2 = [199, 281, 51]
-----END OF CODE ------


1.0
------Code-----
Sign 1  = [CTG, ]
Sign 2  = [CTC, ]
Meaning 1  = [L, ]
Meaning 2  = [T, ]
Codemaker  = [tRNACTCT, tRNACTGL, ]
alt. Codemaker  = [tRNACTCL, tRNACTGT, ]
S1->M1 = [198, 258, 40]
S2->M1 = [201, 238, 40]
S1->M2 = [198, 234, 51]
S2->M2 = [201, 248, 51]
-----END OF CODE ------


1.0
------Code-----
Sign 1  = [CTA, ]
Sign 2  = [CTC, ]
Meaning 1  = [L, ]
Meaning 2  = [T, ]
Codemaker  = [tRNACTAL, tRNACTCT, ]
alt. Codemaker  = [tRNACTAT, tRNACTCL, ]
S1->M1 = [199, 278, 40]
S2->M1 = [201, 238, 40]
S1->M2 = [199, 281, 51]
S2->M2 = [201, 248, 51]
-----END OF CODE ------


1.0
```

```
------Code-----
Sign 1  = [TAA, ]
Sign 2  = [TAG, ]
Meaning 1  = [STOP, ]
Meaning 2  = [Q, ]
Codemaker  = [tRNATAASTOP, tRNATAGQ, ]
alt. Codemaker  = [tRNATAAQ, tRNATAGSTOP, ]
S1->M1 = [220, 250, 44]
S2->M1 = [223, 236, 44]
S1->M2 = [220, 301, 55]
S2->M2 = [223, 245, 55]
-----END OF CODE ------


16 NA 170 1.0
```
