# Supplementary material for: Molecular Codes in Biological and Chemical Reaction Networks
Source: PLoS One. 2013 Jan 23;8(1):e54694. doi: 10.1371/journal.pone.0054694 (PMC3553058; doi:10.1371/journal.pone.0054694)
Supplement: Text S3 — Mathematical model of the phosphorylation cascade shown in Figure 4C . (PDF) [file pone.0054694.s007.pdf]

Model of the phosphorylation cascade shown in Figure 4C.

$$\frac{\mathrm{d}([\mathrm{B}] \cdot V_{\mathrm{cell}})}{\mathrm{d}t} = -V_{\mathrm{cell}} \cdot (0.1 \cdot [\mathrm{S_P}] \cdot [\mathrm{B}]) + V_{\mathrm{cell}} \cdot (0.1 \cdot [\mathrm{B_P}])$$

$$\frac{\mathrm{d}([\mathrm{B_P}] \cdot V_{\mathrm{cell}})}{\mathrm{d}t} = +V_{\mathrm{cell}} \cdot (0.1 \cdot [\mathrm{S_P}] \cdot [\mathrm{B}]) - V_{\mathrm{cell}} \cdot (0.1 \cdot [\mathrm{B_P}])$$

$$\frac{\mathrm{d}([\mathrm{C}] \cdot V_{\mathrm{cell}})}{\mathrm{d}t} = -V_{\mathrm{cell}} \cdot (0.1 \cdot [\mathrm{S_P}] \cdot [\mathrm{C}]) + V_{\mathrm{cell}} \cdot (0.1 \cdot [\mathrm{C_P}])$$

$$\frac{\mathrm{d}([\mathrm{C_P}] \cdot V_{\mathrm{cell}})}{\mathrm{d}t} = +V_{\mathrm{cell}} \cdot (0.1 \cdot [\mathrm{S_P}] \cdot [\mathrm{C}]) - V_{\mathrm{cell}} \cdot (0.1 \cdot [\mathrm{C_P}])$$

$$\frac{\mathrm{d}([\mathrm{A}] \cdot V_{\mathrm{cell}})}{\mathrm{d}t} = -V_{\mathrm{cell}} \cdot (0.1 \cdot [\mathrm{B}] \cdot [\mathrm{A}]) - V_{\mathrm{cell}} \cdot (0.1 \cdot [\mathrm{C_P}] \cdot [\mathrm{A}]) + V_{\mathrm{cell}} \cdot (0.1 \cdot [\mathrm{A_P}])$$

$$\frac{\mathrm{d}([\mathrm{A_P}] \cdot V_{\mathrm{cell}})}{\mathrm{d}t} = +V_{\mathrm{cell}} \cdot (0.1 \cdot [\mathrm{B}] \cdot [\mathrm{A}]) + V_{\mathrm{cell}} \cdot (0.1 \cdot [\mathrm{C_P}] \cdot [\mathrm{A}]) - V_{\mathrm{cell}} \cdot (0.1 \cdot [\mathrm{A_P}])$$

$$\text{"act} - \text{level}_{\mathrm{A}}\text{"} = \frac{[\mathrm{A_P}]}{[\mathrm{A}]}$$

$$\text{"act} - \text{level}_{\mathrm{B}}\text{"} = \frac{[\mathrm{B_P}]}{[\mathrm{B}]}$$

$$\text{"act} - \text{level}_{\mathrm{C}}\text{"} = \frac{[\mathrm{C_P}]}{[\mathrm{C}]}$$
