# Supplementary material for: Molecular Codes in Biological and Chemical Reaction Networks
Source: PLoS One. 2013 Jan 23;8(1):e54694. doi: 10.1371/journal.pone.0054694 (PMC3553058; doi:10.1371/journal.pone.0054694)
Supplement: Text S4 — List of all binary molecular codes (including duplicates) identified in the NTOP chemistry. (PDF) [file pone.0054694.s008.pdf]

Original List of all BMCs (including dublicates) identified in the NTOP chemistry.

```
BMC:  0    1 2    13 15    1 3    8    0 5 10 15    0 1 9

EXPLAIN:  S1 =  1 2   S2 =  13 15
 M1 =   1 3    M2 =  8
 C =  0 10 15 5     C' =  0 1 9
 S1 + C  -->  0 1 10 15 2 3 5
 S2 + C  -->  0 10 12 13 15 5 8
 S1 + C' -->  0 1 2 4 8 9
 S2 + C' -->  0 1 11 13 15 3 5 9

BMC:  1    1 2    13 15    1 3    8    0 10 11 15    0 1 9

EXPLAIN:  S1 =  1 2   S2 =  13 15
 M1 =   1 3    M2 =  8
 C =  0 10 11 15     C' =  0 1 9
 S1 + C  -->  0 1 10 11 15 2 3 5
 S2 + C  -->  0 10 11 12 13 15 8
 S1 + C' -->  0 1 2 4 8 9
 S2 + C' -->  0 1 11 13 15 3 5 9

BMC:  2    1 2    13 15    1 3    8    0 10 15    0 1 9

EXPLAIN:  S1 =  1 2   S2 =  13 15
 M1 =   1 3    M2 =  8
 C =  0 10 15     C' =  0 1 9
 S1 + C  -->  0 1 10 15 2 3 5
 S2 + C  -->  0 10 12 13 15 8
 S1 + C' -->  0 1 2 4 8 9
 S2 + C' -->  0 1 11 13 15 3 5 9

BMC:  3    1 2    13 15    1 3    8    5 10 15    0 1 9

EXPLAIN:  S1 =  1 2   S2 =  13 15
 M1 =   1 3    M2 =  8
 C =  10 15 5     C' =  0 1 9
 S1 + C  -->  1 10 15 2 3 5
 S2 + C  -->  10 12 13 15 5 8
 S1 + C' -->  0 1 2 4 8 9
 S2 + C' -->  0 1 11 13 15 3 5 9

BMC:  4    1 2    13 15    1 3    8    10 11 15    0 1 9

EXPLAIN:  S1 =  1 2   S2 =  13 15
```

```
 M1 =   1 3    M2 =  8
 C =  10 11 15     C' =  0 1 9
 S1 + C  -->  1 10 11 15 2 3 5
 S2 + C  -->  10 11 12 13 15 8
 S1 + C' -->  0 1 2 4 8 9
 S2 + C' -->  0 1 11 13 15 3 5 9

BMC:  5    1 2    13 15    1 3    8    10 15    0 1 9

EXPLAIN:  S1 =  1 2   S2 =  13 15
 M1 =   1 3    M2 =  8
 C =  10 15     C' =  0 1 9
 S1 + C  -->  1 10 15 2 3 5
 S2 + C  -->  10 12 13 15 8
 S1 + C' -->  0 1 2 4 8 9
 S2 + C' -->  0 1 11 13 15 3 5 9

BMC:  6    1 2    13 15    5 15    8    0 10 11 15    0 1 9

EXPLAIN:  S1 =  1 2   S2 =  13 15
 M1 =   15 5    M2 =  8
 C =  0 10 11 15     C' =  0 1 9
 S1 + C  -->  0 1 10 11 15 2 3 5
 S2 + C  -->  0 10 11 12 13 15 8
 S1 + C' -->  0 1 2 4 8 9
 S2 + C' -->  0 1 11 13 15 3 5 9

BMC:  7    1 2    13 15    5 15    8    0 10 15    0 1 9

EXPLAIN:  S1 =  1 2   S2 =  13 15
 M1 =   15 5    M2 =  8
 C =  0 10 15     C' =  0 1 9
 S1 + C  -->  0 1 10 15 2 3 5
 S2 + C  -->  0 10 12 13 15 8
 S1 + C' -->  0 1 2 4 8 9
 S2 + C' -->  0 1 11 13 15 3 5 9

BMC:  8    1 2    13 15    5 15    8    10 11 15    0 1 9

EXPLAIN:  S1 =  1 2   S2 =  13 15
 M1 =   15 5    M2 =  8
 C =  10 11 15     C' =  0 1 9
 S1 + C  -->  1 10 11 15 2 3 5
 S2 + C  -->  10 11 12 13 15 8
 S1 + C' -->  0 1 2 4 8 9
 S2 + C' -->  0 1 11 13 15 3 5 9
```

```
BMC:  9    1 2    13 15    5 15    8    10 15    0 1 9

EXPLAIN:  S1 =  1 2   S2 =  13 15
 M1 =   15 5    M2 =  8
 C =  10 15     C' =  0 1 9
 S1 + C  -->  1 10 15 2 3 5
 S2 + C  -->  10 12 13 15 8
 S1 + C' -->  0 1 2 4 8 9
 S2 + C' -->  0 1 11 13 15 3 5 9

BMC:  10    4 8    11 15    1    8 12    0 8 9    0 5 10 15

EXPLAIN:  S1 =  4 8   S2 =  11 15
 M1 =   1    M2 =  12 8
 C =  0 8 9     C' =  0 10 15 5
 S1 + C  -->  0 1 2 4 8 9
 S2 + C  -->  0 10 11 12 13 15 8 9
 S1 + C' -->  0 10 12 15 4 5 8
 S2 + C' -->  0 1 10 11 15 3 5

BMC:  11    4 8    11 15    1    8 12    8 9    0 5 10 15

EXPLAIN:  S1 =  4 8   S2 =  11 15
 M1 =   1    M2 =  12 8
 C =  8 9     C' =  0 10 15 5
 S1 + C  -->  1 2 4 8 9
 S2 + C  -->  10 11 12 13 15 8 9
 S1 + C' -->  0 10 12 15 4 5 8
 S2 + C' -->  0 1 10 11 15 3 5

BMC:  12    4 8    11 15    1    10 15    0 8 9    0 5 13 15

EXPLAIN:  S1 =  4 8   S2 =  11 15
 M1 =   1    M2 =  10 15
 C =  0 8 9     C' =  0 13 15 5
 S1 + C  -->  0 1 2 4 8 9
 S2 + C  -->  0 10 11 12 13 15 8 9
 S1 + C' -->  0 10 12 13 15 4 5 8
 S2 + C' -->  0 1 11 13 15 3 5

BMC:  13    4 8    11 15    1    10 15    8 9    0 5 13 15

EXPLAIN:  S1 =  4 8   S2 =  11 15
 M1 =   1    M2 =  10 15
 C =  8 9     C' =  0 13 15 5
```

```
 S1 + C  -->  1 2 4 8 9
 S2 + C  -->  10 11 12 13 15 8 9
 S1 + C' -->  0 10 12 13 15 4 5 8
 S2 + C' -->  0 1 11 13 15 3 5

BMC:  14    5 15    9    1    8 12    0 10 11 15    0 4 8

EXPLAIN:  S1 =  15 5   S2 =  9
 M1 =   1    M2 =  12 8
 C =  0 10 11 15     C' =  0 4 8
 S1 + C  -->  0 1 10 11 15 3 5
 S2 + C  -->  0 10 11 12 13 15 8 9
 S1 + C' -->  0 10 12 15 4 5 8
 S2 + C' -->  0 1 2 4 8 9

BMC:  15    5 15    9    1    8 12    10 11 15    0 4 8

EXPLAIN:  S1 =  15 5   S2 =  9
 M1 =   1    M2 =  12 8
 C =  10 11 15     C' =  0 4 8
 S1 + C  -->  1 10 11 15 3 5
 S2 + C  -->  10 11 12 13 15 8 9
 S1 + C' -->  0 10 12 15 4 5 8
 S2 + C' -->  0 1 2 4 8 9

BMC:  16    9    10 15    1 3    8    0 5 13 15    0 1 2

EXPLAIN:  S1 =  9   S2 =  10 15
 M1 =   1 3    M2 =  8
 C =  0 13 15 5     C' =  0 1 2
 S1 + C  -->  0 1 11 13 15 3 5 9
 S2 + C  -->  0 10 12 13 15 5 8
 S1 + C' -->  0 1 2 4 8 9
 S2 + C' -->  0 1 10 15 2 3 5

BMC:  17    9    10 15    1 3    8    5 13 15    0 1 2

EXPLAIN:  S1 =  9   S2 =  10 15
 M1 =   1 3    M2 =  8
 C =  13 15 5     C' =  0 1 2
 S1 + C  -->  1 11 13 15 3 5 9
 S2 + C  -->  10 12 13 15 5 8
 S1 + C' -->  0 1 2 4 8 9
 S2 + C' -->  0 1 10 15 2 3 5
```

```
unique mappings, all mappings, closures:
6 18 244
```
